# Numerical Simulation on Slabs Dislocation of Zipingpu Concrete Faced Rockfill Dam during the Wenchuan Earthquake Based on a Generalized Plasticity Model

**DOI:** 10.1155/2014/572407

**Published:** 2014-06-09

**Authors:** Bin Xu, Yang Zhou, Degao Zou

**Affiliations:** ^1^The State Key Laboratory of Coastal and Offshore Engineering, Dalian University of Technology, Dalian 116024, China; ^2^Institute of Earthquake Engineering, Dalian University of Technology, Dalian 116024, China

## Abstract

After the Wenchuan earthquake in 2008, the Zipingpu concrete faced rockfill dam (CFRD) was found slabs dislocation between different stages slabs and the maximum value reached 17 cm. This is a new damage pattern and did not occur in previous seismic damage investigation. Slabs dislocation will affect the seepage control system of the CFRD gravely and even the safety of the dam. Therefore, investigations of the slabs dislocation's mechanism and development might be meaningful to the engineering design of the CFRD. In this study, based on the previous studies by the authors, the slabs dislocation phenomenon of the Zipingpu CFRD was investigated. The procedure and constitutive model of materials used for finite element analysis are consistent. The water elevation, the angel, and the strength of the construction joints were among major variables of investigation. The results indicated that the finite element procedure based on a modified generalized plasticity model and a perfect elastoplastic interface model can be used to evaluate the dislocation damage of face slabs of concrete faced rockfill dam during earthquake. The effects of the water elevation, the angel, and the strength of the construction joints are issues of major design concern under seismic loading.

## 1. Introduction

A large earthquake (*M*s = 8.0) occurred on May 12, 2008, in Wenchuan, Sichuan Province, China. After Wenchuan earthquake, field investigations showed that there were extensive dislocations of face slabs along construction joints between the second and third stages. The phenomenon of dislocation of face slabs was previously summarized [1, 2]. The damage mechanism of Zipingpu CFRD was also analyzed [1] based on field investigation. It was concluded that dislocations of face slabs were due to the permanent deformation of the dam and the lower strength of the construction joints as compared to the concrete slabs. However, most of these summaries are phenomenological in nature. There have been few numerical studies until now because of the limit of the rockfill constitutive model development.

So far the equivalent linear analysis based on viscoelastic constitutive models [3] is the main method used for the dynamic response analysis of high CFRDs [4–6]. However, the deformation calculated by the equivalent linear analysis was elastic deformation and returned to zero at the end of earthquake. That is to say, the seismic residual deformation of the dam, which is important for the seismic design of high CFRDs, cannot be obtained directly by this method, especially the deformation history. To cover the shortage, two approximate approaches are usually used to evaluate the residual deformation of dams additionally. One is the limit equilibrium method for rigid block—Newmark sliding block analysis [7]—based on the yield acceleration concept and the other one is the global deformation method based on the strain potential concept [8]. However, in the above two approaches, dam's dynamic response analysis and residual deformation calculation process are separated artificially. In fact, the residual deformation mainly occurred during the earthquake.

Therefore, developing the elastic-plastic model of the rockfill materials and related analysis procedure is important for the CFRD dynamic analysis and earthquake damage simulation. Based on the generalized plasticity theory [9, 10], the authors developed an elastic-plastic constitutive model of the rockfills. The model could consider the pressure dependency of rockfill materials under loading, unloading, and reloading conditions and was used to simulate the construction process and the dynamic responses of the Zipingpu CFRD during Wenchuan earthquake successfully [11, 12].

In this paper, the validated dynamic finite element procedure was used to conduct a series of studies on the 3D dynamic analysis in gaining additional insight into the effects of water elevation, angle, and strength of the construction joints on the performance of the slabs dislocation during earthquake loading. The major results of study are summarized and discussed.

## 2. Slabs Dislocation of Zipingpu Dam during Wenchuan Earthquake

**Figure 1:**
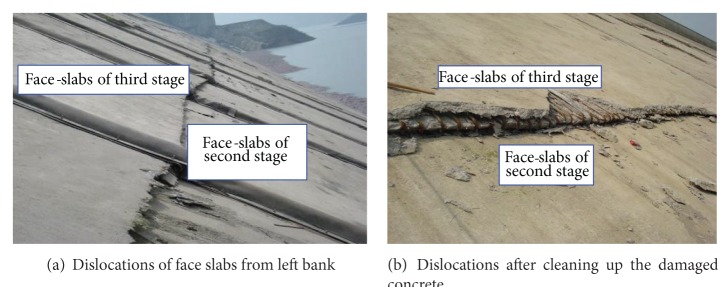
Dislocations of face slabs along construction joints between second and third stages.

Zipingpu CFRD was obviously damaged during Wenchuan earthquake [1, 2]. Detailed information of the dam has been previously provided by the authors [12]. Serious dislocation damage also occurred between stages II and III slabs at EL. 845 m, as shown in Figure 1. The steel rebar in the concrete slabs was bended to “Z” shape (Figure 1(b)). Concrete cracked and fell off below the construction joints at EL. 845.

## 3. Constitutive Model

### 3.1. Generalized Plasticity Model Modified for Rockfills

In this study, the modified generalized model was used for the rockfill materials, and a perfect elastoplastic interface model with pressure-dependent shear stiffness was employed to simulate the interfaces between the face slabs and cushion gravel. For further details, refer to [11, 12]. This paper focuses on the slabs dislocation of the dam during strong earthquakes.

## 4. Parameters Identification

### 4.1. Rockfill Materials

The rockfill material parameters are provided in Table 1 [12]. The model parameters are consistent with those used in the simulation of the construction process of the Zipingpu CFRD, and the capacity of the constitutive model in describing the virgin loading, unloading, and reloading and cyclic loading responses of the Zipingpu rockfill material is demonstrated in the previous paper [11, 12].

### 4.2. Interface

The interfaces between the concrete slabs and cushion gravel were experimentally investigated by Zhang and Zhang [13, 14]. The perfect elastoplastic interface parameters were calibrated using their test results and are listed in Table 2 [12].

### 4.3. Vertical Slab Joints and Peripheral Joints

The linear elastic interface model was used for slab joints and peripheral joints. The parameters used in the dynamic analysis were consistent with those in the previous study [12].

### 4.4. Construction Joints

In this study, the linear elastic interface model was also used for construction joints and the constructions were simulated based on the reduced shear strength of concrete, the parameters are the same with that used in [11, 12]. The dynamic shear strength of the construction joints was assumed to be 0.545 MPa in the dynamic analysis and 1.365 MPa in the static simulation.

## 5. Finite Element Analysis

### 5.1. 3D FE Program

With the developed elastic-plastic model of the rockfill and interface model mentioned above, using the object-oriented programming method, the authors completed a three-dimensional finite element program—geotechnical nonlinear dynamic analysis (GEODYNA) [15]. The GEODYNA program was used to simulate the construction process and dynamic responses of the Zipingpu CFRD successfully.

### 5.2. FE Mesh

The same 3D finite element mesh of the Zipingpu CFRD used in the construction process simulation was adopted for the dynamic analysis [12]. In total, 23,994 elements were included in the mesh, including 614 slab elements. Finally, the hydrodynamic pressure acting on the face slabs was simulated in the dynamic analysis using the adding mass method [16]; the mass element was defined by single node, concentrated mass components. There are 317 added mass elements in total.

### 5.3. Input Ground Motions

**Figure 2:**
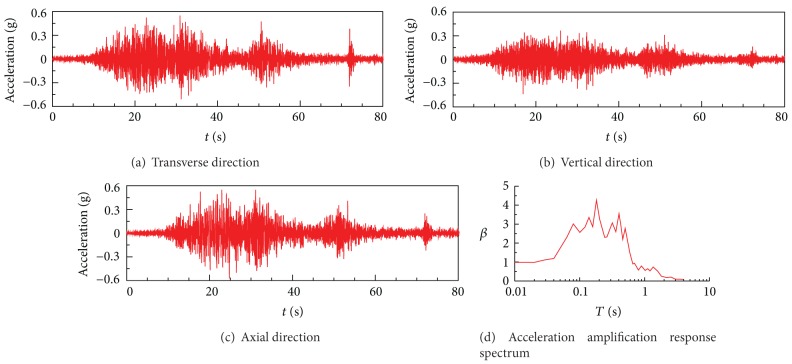
Input earthquake motion.

As no bedrock acceleration time histories were recorded at the dam site during Wenchuan earthquake, bedrock acceleration time histories measured at Mao Town, which is located 75 km from Zipingpu Dam, were adopted as input ground motions [17]. The acceleration time histories and acceleration response spectrum are shown in Figure 2. The measured horizontal acceleration time history at Mao Town was scaled to have a PGA of 0.55 g and the vertical one was assumed to be 2/3 of the horizontal.

## 6. Results and Discussions

The dynamic responses of the Zipingpu CFRD, including the acceleration and the dam settlement during earthquake, were described in the previous study [12]. In this paper, the slabs dislocation and its main influenced factors would be discussed mainly.

### 6.1. Water Elevation

**Figure 3:**
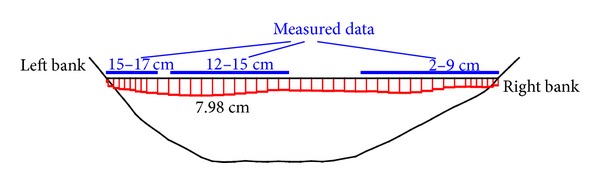
The simulated dislocation distribution of the construction joints between stages II and III slabs at the end of the earthquake.

**Figure 4:**
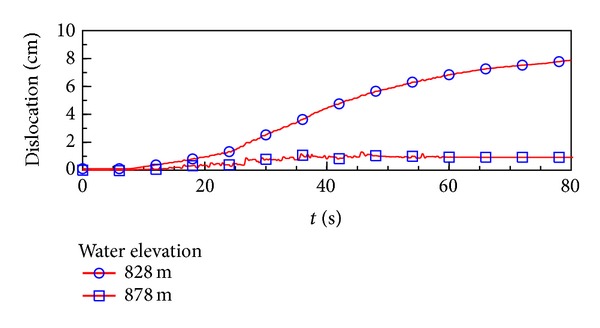
The slab dislocation development during earthquake with different water elevations.

During the Wenchuan earthquake, the reservoir water elevation was at 828.76 m, which was lower than the construction joint elevation (EL. 845 m) between stages II and III slabs. In this study, the water elevation was taken as EL. 828 m and 878 m (full reservoir elevation) to study its influence. The simulated dislocation distribution of the construction joints between stages II and III slabs with the water elevation of EL. 828 m, at the end of the earthquake shaking, is illustrated in Figure 3, and the maximum dislocation reached 7.98 cm. Though the maximum simulated dislocation was less than the measured dislocation (17 cm), the distribution is similar. This difference of dislocation value could be because only shear failure was considered in this study, while in reality tensile damage may have contributed to the dislocation due to the separation of the slabs from the cushion layers.  Figure 4 reveals the development of the dislocation history of the construction joint element in which the maximum dislocation occurred during earthquake shaking with different water elevations. When the water elevation reached EL. 878 m, the maximum dislocation displacement was only 0.91 cm. It indicates that the water elevation has a greater impact on the dislocation of face slab. This indicated that water pressure supported the upper part of the dam when the water elevation was higher than the joint elevation. Without the support, the face slabs dislocation would develop more easily.

However, it should be noted that even less dislocation when the water elevation was higher than the joint elevation, it is very dangerous since seepage would have occurred and affected significantly the dam's safety. It is therefore concluded that face slabs of CFRD need to be reinforced at the construction joints, using measures such as stretching rebar buried in the top of the dam, in order to increase the seismic resistance.

### 6.2. Angle of the Construction Joints

**Figure 5:**
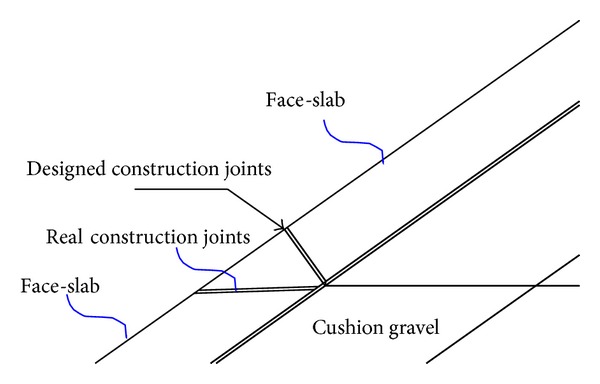
Diagram of the angle of the construction joints.

**Figure 6:**
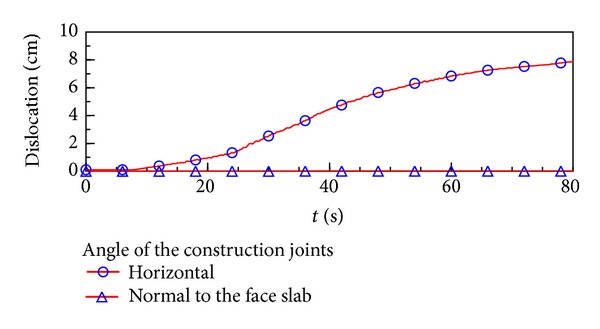
The slabs dislocation development during earthquake with different construction joints angles.

As shown in Figure 5, the angle of the construction joints was designed normal to the face slab. However, it was constructed to be horizontal. When the water elevation is EL. 828 m, the development of the development history of the typical construction joint element during earthquake shaking with different water elevation was illustrated as shown in Figure 6. With the angle being normal to the face slab, the maximum dislocation is only 0.11 cm, which is reduced greatly compared with the horizontal angle.

### 6.3. Dynamic Strength of the Construction Joints

As mentioned in the section of parametric identification, the dynamic shear strength of the construction joints was considered to be lower than the whole casting concrete. In the above analysis, the dynamic shear strength of the construction joints was assumed as 0.545 MPa. In this section, the dynamic strength of the construction joints was taken as 0.545 MPa, 1 MPa, 2 MPa, and 2.73 MPa (without construction joints) to research its impact on the slabs dislocation.

**Figure 7:**
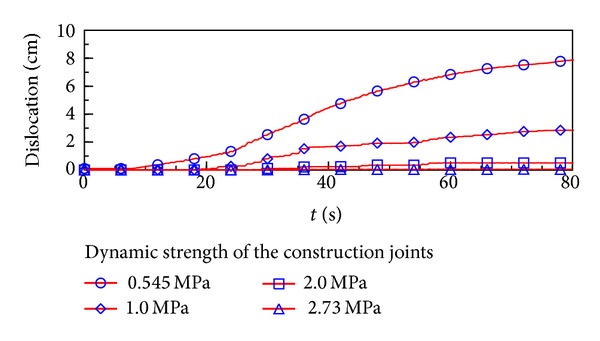
The slabs dislocation development during earthquake with different dynamic strengths of construction joints.

Figure 7 illustrated the development of the typical construction joint element dislocation with different dynamic strengths during earthquake. The dislocation value decreased from 7.98 cm to almost zero, while the dynamic strength increased from 0.545 MPa to 2.73 MPa. It could be concluded that measures enhancing the dynamic strength of the construction joints would control the slabs dislocation effectively, such as increasing the reinforcement ratio.

## 7. Discussion and Conclusions

The Zipingpu dam is the highest CFRD over 150 m to be subjected to strong earthquake shaking, and the slabs dislocation is one of the typical damage phenomena not found in previous earthquake. This event provides a rare opportunity to verify the seismic design and safety evaluation procedures for high CFRDs. The 3D dynamic response of the Zipingpu dam during the Wenchuan earthquake was simulated based on a modified generalized plasticity model for rockfill materials in gaining additional insight into the effects of water elevation, angle, and strength of the construction joints on the performance of the slabs dislocation during earthquake loading.

The dislocation phenomenon that occurred during the Wenchuan earthquake was successfully captured by the proposed numerical procedure; however, the calculated magnitude was smaller than the measured magnitude. This discrepancy may have arisen from the linear elastic assumption of the concrete slabs and the simple shear failure model of construction joints employed in this study.

The effects of the water elevation, the angle, and the strength of the construction joints are issues of major design concern under seismic loading. Reinforcement at the construction joints should be enhanced to restrain the dislocation damage of the slabs. Furthermore, the angle of the construction joints should be designed and constructed as normal to the slabs, which will also decrease the slabs dislocation to a great extent.

## Figures and Tables

**Table 1:** Rockfill material parameters in the modified generalized plasticity model.

*G*_0_	*K*_0_	*M*_*g*_	*M*_*f*_	*α*_*f*_	*α*_*g*_	*H*_0_	*H*_*U*0_	*m*_*s*_	*m*_*v*_	*m*_*l*_	*m*_*u*_	*r*_*d*_	*γ*_DM_	*γ*_*u*_	*β*_0_	*β*_1_
1000	1400	1.8	1.38	0.45	0.4	1800	3000	0.5	0.5	0.2	0.2	180	50	4	35	0.022

**Table 2:** Parameters of the concrete-gravel interfaces of the Zipingpu CFRD.

*k*_1_	*k*_2_	*n*	*φ*	*c*
300	1e10	0.8	41.5	0
